# “We are not stray leaves blowing about in the wind”: exploring the impact of Family Wellbeing empowerment research, 1998–2021

**DOI:** 10.1186/s12939-021-01604-1

**Published:** 2022-01-10

**Authors:** Nirukshi Perera, Komla Tsey, Marion Heyeres, Mary Whiteside, Leslie Baird, Janya McCalman, Yvonne Cadet-James, Bianca Calabria, Michael Hamilton, Li Yan, Ines Zuchowski, Kearrin Sims, Hyacinth Udah

**Affiliations:** 1 Resuscitation & Emergency Care Research Unit, Curtin University, Bentley, WA Australia; 2 The Cairns Institute, James Cook University, Smithfield, QLD Australia; 3 Office of Allied Health, Human Services & Sport, La Trobe University, VIC, Australia; 4 Centre for Indigenous Health Equity Research, School of Health, Medical and Applied Sciences, Psychology and Public Health Department, Central Queensland University, Cairns, QLD Australia; 5 Indigenous Research and Education Centre, James Cook University, Townsville, QLD Australia; 6 College of Health and Medicine, Australian National University, Canberra, ACT Australia; 7 Batchelor Institute of Indigenous Tertiary Education, Batchelor, NT Australia; 8 College of Economics and Management, Shenyang University of Chemical Technology, Shenyang, China; 9 College of Arts, Society and Education, James Cook University, Townsville, QLD Australia; 10 College of Arts, Society and Education, James Cook University, Smithfield, QLD Australia

**Keywords:** Aboriginal and Torres Strait Islander health, Indigenous health, Empowerment, Research impact, Social and emotional wellbeing, Research evaluation, Logic model, Theory of change, Implementation science, Service utilisation

## Abstract

**Background:**

An Aboriginal-developed empowerment and social and emotional wellbeing program, known as Family Wellbeing (FWB), has been found to strengthen the protective factors that help Indigenous Australians to deal with the legacy of colonisation and intergenerational trauma. This article reviews the research that has accompanied the implementation of the program, over a 23 year period. The aim is to assess the long-term impact of FWB research and identify the key enablers of research impact and the limitations of the impact assessment exercise. This will inform more comprehensive monitoring of research impact into the future.

**Methods:**

To assess impact, the study took an implementation science approach, incorporating theory of change and service utilisation frameworks, to create a logic model underpinned by Indigenous research principles. A research impact narrative was developed based on mixed methods analysis of publicly available data on: 1) FWB program participation; 2) research program funding; 3) program outcome evaluation (nine studies); and 4) accounts of research utilisation (seven studies).

**Results:**

Starting from a need for research on empowerment identified by research users, an investment of $2.3 million in research activities over 23 years produced a range of research outputs that evidenced social and emotional wellbeing benefits arising from participation in the FWB program. Accounts of research utilisation confirmed the role of research outputs in educating participants about the program, and thus, facilitating more demand (and funding acquisition) for FWB. Overall research contributed to 5,405 recorded participants accessing the intervention. The key enablers of research impact were; 1) the research was user- and community-driven; 2) a long-term mutually beneficial partnership between research users and researchers; 3) the creation of a body of knowledge that demonstrated the impact of the FWB intervention via different research methods; 4) the universality of the FWB approach which led to widespread application.

**Conclusions:**

The FWB research impact exercise reinforced the view that assessing research impact is best approached as a “wicked problem” for which there are no easy fixes. It requires flexible, open-ended, collaborative learning-by-doing approaches to build the evidence base over time. Steps and approaches that research groups might take to build the research impact knowledge base within their disciplines are discussed.

**Supplementary Information:**

The online version contains supplementary material available at 10.1186/s12939-021-01604-1.

## Background

Improving empowerment and social and emotional wellbeing (SEWB) among Aboriginal and Torres Strait Islander^1^ people is critical for addressing health and wellbeing inequity. In Australia, there exists a trans-generational legacy of complex trauma associated with colonisation, dispossession, inflexible systems and government policies, such as the removal of children from their families [1]. This has resulted in many young Indigenous people living in situations of severe social adversity [2, 3]. A broad range of indicators evidence legacies of colonisation, with young Indigenous people reported to be: seven times as likely to receive child protection services; 26 times as likely to be in the juvenile justice system; 2.7 times as likely to be unemployed; and three times as likely to live in overcrowded housing compared to young non-Indigenous people [4–8]. Underlying this inequity is the fact that First Nations people never ceded their sovereignty over the diverse lands that make up Australia, yet successive governments are yet to meaningfully commit to the idea of a treaty [9].

To overcome the effects of these problems, individuals and communities need to build protective factors such as connection to family, country, language and culture. Empowerment enables people to have more control over their lives and the social and economic environment in which they live [1]; and has been found to improve education and employment outcomes for Aboriginal people in remote Australia [9].

The persistence of alarming inequities between Indigenous and non-Indigenous Australians also places urgency on research to help build the evidence base for protective factors; to be beneficial to the researched; to be accountable; and to address needs identified by Indigenous people [10]. As a prominent leader in Indigenous health research, Dr Pat Anderson, powerfully expressed, “we need to have a sense of agency in our lives, that we are not stray leaves blowing about in the wind … we need empowerment” [11]. Not only is there a need for interventions that support this goal, but their impact on SEWB must be monitored and the research findings must be transferred to the relevant parties so that those parties can make informed decisions. This has great potential to improve health equity.

However, reviews of SEWB (which included empowerment) interventions for Indigenous people have found that few programs are documented in the literature and few have been evaluated [12]. Of those evaluated, the dearth of rigorous research designs and validated quantitative measures has attracted a certain stigma because of a perceived lack of specificity or quantification. In addition, a systematic review of Indigenous health research found little reporting of the impact of such research for Aboriginal and Torres Strait Islander people in Australia [13], and this is less so when it comes to reporting impact on difficult-to-quantify constructs such as empowerment and wellbeing [14]. Internationally, there is a lack of reporting on research impact, that is, how the evidence translates into meaningful changes. There is a need for clarity on how to report research impact; for understanding the enablers of impact; and for transparent impact reporting [15].

Research impact is defined as “the demonstrable contribution that research makes to the economy, culture, national security, public policy or services, health, the environment, or quality of life, beyond contribution to academia” [16]. The expectation of researchers to demonstrate this impact, in addition to the usual quality measures such as journal impact and citation frequency, is growing [14]. This is evidenced by the UK government’s introduction of a Research Impact Assessment framework in 2014 and the Australian Research Council’s (ARC) three-yearly Engagement and Impact Assessment in 2018 [15, 17]. While this focus is relatively new in academic and government settings, Indigenous Australian community leaders and organisations have been calling for scholars to be held accountable for their impact and to identify how their work enhances health equity for Indigenous people for some time [14, 18–21]. This article explores the impact of a research program, based on an SEWB and empowerment intervention called Family Wellbeing (FWB), which has spanned 23 years and has involved the development of a national network of researchers and communities.

The task of reporting health research impact is complex with several methodological challenges. Firstly, there can be a significant time lag between conducting research and then using the results to translate into change for participants. It is also difficult to draw a clear cause and effect relationship due to the many factors that combine to produce change. Furthermore, researchers must mine retrospective data, that were collected for various purposes, to build an argument that impact occurred. Tracking societal impact also involves a time and resource cost that exceeds the relative immediacy of providing bibliometric measures such as citations [18, 21–24]. However if researchers do not attempt to assess real impact, even through limited retrospective data, then there is risk that the impact agenda becomes no more than box-ticking rather than a genuinely reflexive exercise [14].

When the ARC piloted their research impact assessment framework in 2017, it was a catalyst for the FWB research network to systematically explore its research impact. The ARC’s requirements to report impact according to one’s current institutional affiliation and within a 15-year time frame were examined; along with the constraints this put on assessing a 23-year research program in which the key researchers changed institutional affiliations over time [14]. The feasibility of using the framework was also explored to describe FWB research impact in one specific community and the challenges and opportunities involved [25].

This article, therefore, builds on the two aforementioned studies to take a long-term view of FWB research impact and to enable planning and collection of appropriate data that can support such impact claims. The aims of this article are to: 1) use retrospective data to explore the impact of a 23-year long research program; 2) identify the key factors that have enabled research impact; 3) identify any limitations in the impact data to provide a way forward for improving the research impact evidence-base.

In the next two sections, a brief description of the FWB program and associated research to date is given. Since the focus of the article is on exploring the impact of research about the FWB program, it is important to distinguish between the impact of the empowerment intervention called FWB and the research that accompanied the journey of the intervention. Therefore, the former is referred to as “FWB program outcomes” and the latter as “FWB research impact”.

### Family Wellbeing program description

FWB is an empowerment program developed by Aboriginal Australians (via the Aboriginal Education Development Branch of the South Australian Department of Education) in 1993. It aims to empower Indigenous people to deal with the after-effects of colonisation, and other problems associated with rapid social change, by helping individuals, families and communities to take greater control and responsibility over their lives. FWB is an accredited program through the Australian vocational educational training system, comprising 150 h of facilitated small group learning (full version). It can also be modularised to comprise just one 30-h introductory component (foundation version).

FWB workshops exemplify a trauma-informed approach to healing. Topics covered include human qualities; basic human needs (physical, emotional, mental and spiritual); human relationships; life journey; beliefs and attitudes; violence and abuse; addictions; crisis and emotions; loss and grief; conflict resolution; and caring for ourselves and others [11, 26, 27]. The goal is to strengthen the protective factors needed to take greater control of their situation despite the legacy of colonisation and health inequity.

Although FWB was developed primarily in response to the special needs of Indigenous Australians, it can be adaptable to the needs of other cultures and social groups in Australia and beyond [28–31].

### Family Wellbeing research to date

**Fig. 1:**
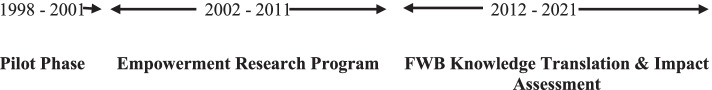
Overview of FWB research and translation activities: Three broad overlapping phases

This section summarises the journey of FWB research over three overlapping phases (Fig. 1).

#### Phase 1

Phase 1 encompassed the pilot program evaluation which took place between 1998 and 2001. In this phase, FWB addressed needs that were identified as a priority by the Indigenous participants in the program, namely empowerment and SEWB as protective factors against widespread trauma and disadvantage. The program’s explicit foci on suicide prevention, family violence, parenting, and employment readiness had long been identified as critical to improving Indigenous health, but there had been little reporting about practical interventions to address these needs [27].

The pilot evaluation adopted a narrative approach based on participants’ advice to “ask us to tell stories or ask us to write our stories of change in diaries” [27]. This approach facilitated a strengths-based evaluation that prioritised Aboriginal people’s views and experiences. The Cooperative Research Centre for Aboriginal and Tropical Health (CRCATH) (1997–2002) distributed the evaluation report to Indigenous organisations widely, resulting in more interest and ultimately demand for the FWB program.

#### Phase 2

Phase 2 covered a 10-year program of research known as the Empowerment Research Program (ERP) [11, 32, 33]. The ERP had a broad research agenda in which systematically exploring the potential contribution of FWB to empowerment and improving Indigenous SEWB was a key part. The research group drew upon a combination of Indigenous research principles (Indigenous leadership and participation; addressing priority needs; being beneficial; transferring benefits to others; enhancing capacity [10, 26]); and a “phased-approach”, including both short- and long-term objectives, to evaluating complex interventions [32, 34]. Evaluation of the FWB program confirmed its applicability in a variety of contexts such as community wellbeing promotion, school health promotion, and workforce development; and that the consistent outcome of participation in FWB was individuals’ enhanced capacity to exert greater control over factors shaping their SEWB [35].

A close relationship with the Lowitja Institute, Australia’s national institute for Aboriginal and Torres Strait Islander health research, and the associated co-operative research centres that predated it (i.e. the CRCATH & the Cooperative Research Centre for Aboriginal Health), assisted the impact of FWB research. Indigenous researchers associated with the Institute provided expertise that was integrated into the research program [11]. The Lowitja Institute supported FWB research translation activities including funding FWB research policy briefs, FWB knowledge sharing forums, discussion papers, and plain language community reports [36–38]. By the end of Phase 2 in 2011, FWB had been introduced, with varying degrees of sustainability, in 57 sites around Australia [39].

#### Phase 3

In Phase 3, the research findings from Phases 1 and 2 enabled a focus on knowledge translation and impact assessment which included supporting further uptake of the program. The FWB research network collaborated with Indigenous service providers to start embedding FWB within their core services and programs such as for Indigenous child protection and family support in north Queensland [1] and social and emotional wellbeing in rural Victoria [40].

## Methods

The overall aims of this study were to explore the long-term impact of FWB research and the key enablers of such impact in order to provide a way forward for improving the research impact evidence-base. This involved the following methodological approach and data collection.

### Approach

The study framework drew upon implementation science approaches including theory of change and service utilisation to explore the enablers of FWB research impact. Most implementation science related models focus on understanding the enablers of research or service uptake by ensuring a suitable fit between the implementation of the research, the actual intervention proposed for uptake, and the structural, organisational and individual contexts involved [39]. However, there is limited knowledge about how implementation science approaches could be applied to research/service utilisation for minority Indigenous populations in developed countries. In the contexts of colonised Indigenous populations such as for Aboriginal and Torres Strait Islander people in Australia, implementation and utilisation frameworks need to be significantly modified to take account of changing power relations between Indigenous and other Australians and the right and assertion of Indigenous peoples to take greater control and ownership of research and/or services intended for their benefits [39]. Hence the implementation science approach had to be explicitly grounded in Indigenous ethical research principles such as Indigenous control and ownership of research, including leadership and participation; capacity enhancement; benefit and transferring such benefits and lessons to others. This approach meant that tracking impact was based on progress towards achieving the intended outcomes of the research, as defined by Indigenous people [41].

The theory of change was mapped out in a logic model to provide, in diagrammatic form, the rationale that links the population to be serviced, with the values and strategies believed to lead to desired outcomes, and the actual outcomes [42]. The logic model is predictive and linear, positing that if the first block is in place, then subsequent blocks are likely to follow on from that and reach the desired outcome. Such a model is limited in its ability to reflect complex social conditions and research pathways [24], however it plays an important role in approximating the potential path for research translation and impact [21].

### Data and analysis

For data analysis, as previously noted, the authors first mapped out the FWB research impact theory of change, in the form of a logic model. This showed the pathways through which participation in FWB training workshops resulted in improved SEWB for participants (FWB program outcomes), and the extent to which knowledge of research confirming this finding influenced further program uptake and spread among user organisations and communities (FWB research impact). Based on the impact logic, an overarching research impact narrative was developed showing the scope and extent of the SEWB outcomes, the beneficiaries, and the relative contribution of the James Cook University (JCU)-led 23-year program of FWB research to the impact claim. In order to evidence the impact claim, we concentrated on four main areas for data collection: 1) participation; 2) funding; 3) program outcome evaluation; and 4) accounts of research utilisation.

Data collection deliberately drew on publicly available sources to avoid the cost of collecting new impact data and to ensure public access to verifiable data (See Additional file 1). We searched the online institutional repository for JCU [43] and the National Centre for FWB website [44]. We also searched the *Research Achievements, Empowerment Research Program 2001 – 2020* database, maintained by our network, which contains all available data about FWB research inputs, activities, outputs and evidence of research uptake and subsequent impacts [14]. These sources provided details of all peer-reviewed papers (n = 49); research reports (n = 19); participant numbers; grants and funding sources; DVDs; videos and documentary films; training packages; citations of FWB research in government and other policy documents; policy briefs; and FWB newsletters. Other outputs included one book, four book chapters and three doctoral theses.

We collated FWB participant data from the three FWB program training providers; Technical and Further Education, South Australia (TAFE SA), Batchelor Institute of Indigenous Tertiary Education (Northern Territory (NT)), and JCU-led empowerment research network, up until the year 2011. Post-2011, sole participant data source has been the JCU-led research network as data from other sources was not available.

To develop the FWB research impact narrative, the FWB participation data was analysed, using descriptive statistics, to show the growth in participation and the relative contribution of the JCU-led research network which delivered the program as well. Funding data was analysed, using descriptive statistics, to show the scope and trends in funding or investment in FWB research over time. While this paper is focused on research impact, it is necessary to show what the research found. Therefore, a systematic scoping review of the nine FWB evaluative studies was conducted to show the reported SEWB benefits arising from individuals attending FWB training workshops. This was followed by a systematic scoping review of the seven FWB research translation resources to show the extent to which our research outputs influenced the decisions of service providers and other FWB user organisations to take up FWB and make it available to their clients and service communities.

## Results

The results section is divided into the five steps outlined in our approach to analysis. First, the research program impact logic is presented along with a brief impact narrative and statement of the relative contribution of the research. This leads to an overview of participation in the FWB program over 23 years. Then, trends in FWB research funding and investment will be outlined to show how the research network was enabled to undertake research on SEWB impacts of participation in the FWB program. After that, findings from the accounts of how FWB research was utilised by user organisations and public bodies are summarised. Following these results, a discussion section will highlight the key enablers of research impact, the limitations of tracking such impact, and the plan towards improving the FWB research impact evidence-base.

### Family Wellbeing research impact logic model and overall impact claim

**Fig. 2:**
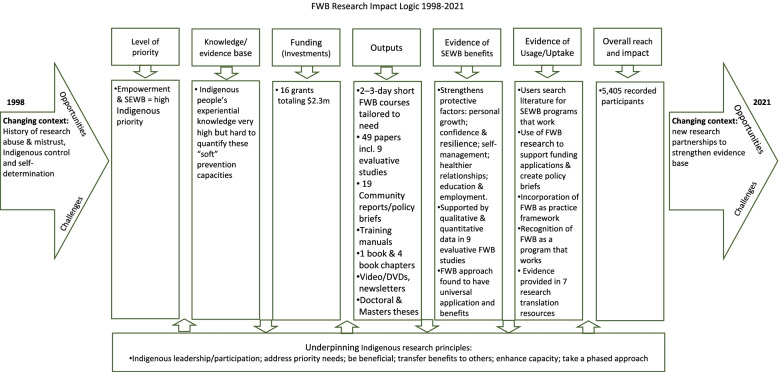
Family Wellbeing research impact logic model

The FWB research impact logic model is presented in Fig. 2 to depict the key enablers of FWB research impact.

As the left-hand side arrow in Fig. 2 shows, planning the pathways through which the FWB intervention and research program lead to impact requires considering the social, economic, political and historical context in which research is being done [45]. When the research began in 1998, there were challenges such as a history of research abuse in Indigenous communities resulting in mistrust of research. At the same time, there were opportunities in the form of enthusiasm for Indigenous control over research, which also tended to put pressure on the small number of Indigenous researchers who had to try and meet competing demands.

The first block in the logic model identifies the priorities for research. The need for research on SEWB and empowerment came from Indigenous community organisations rather than being researcher driven. At that point in time, twenty or so years ago, reports such as the National Aboriginal Health strategy, Ways Forward (National Consultancy Report on Aboriginal and Torres Strait Islander Mental Health) and Bringing Them Home (National Inquiry into the Separation of Aboriginal and Torres Strait Islander Children) all highlighted the need to apply an intergenerational trauma lens and look at aspects such as empowerment and healing. While people had instinctive knowledge of these concepts, it was a challenge for frontline workers to operationalise such ideas within their organisations. Therefore there was a call to understand how to practically enhance SEWB [27] and FWB, as an intervention, offered an Aboriginal-designed program for empowerment.

Aboriginal organisations, and the Indigenous communities they were situated in, approached researchers to do the research on SEWB rather than the other way around (the second block in the logic model). Researchers had to think about the evidence base for SEWB. While there was a lot of experiential knowledge, such concepts were difficult to quantify, and solely descriptive and qualitative findings would not necessarily convince funding bodies of the value of FWB interventions. The research program had a long-term goal to build on the qualitative research by developing quantitative SEWB outcome measures. In accordance, the network developed into long term collaborative research partnerships spanning two decades. This involved Indigenous leadership and participation, for example, co-leadership of the research program by Yvone Cadet-James, an Indigenous academic, and Komla Tsey, a non-Indigenous academic. The research was nested within Indigenous controlled research organisations such as The Lowitja Institute; and there was an emphasis on growing an Indigenous FWB facilitation and research workforce.

These previous aspects of the research program allowed for $2.3 million funding to be attracted and invested as shown in the inputs (investments) block, and this enabled a variety of research outputs (the fourth block). The research network has produced 49 peer-reviewed articles including theoretical papers which explain how FWB participants experience empowerment; whether FWB is accepted and feasible in different Indigenous contexts; how to evaluate FWB outcomes qualitatively and quantitatively; and the costs of implementing the FWB intervention. Importantly, Indigenous team members, who have not had the opportunity to go through the conventional research pathways, published research papers where they reflected, not only on the wellbeing benefits experienced as FWB program participants, but on the challenges and opportunities involved in becoming FWB facilitators and researchers [36, 46–48]. Taking guidance from research participants, narrative storytelling approaches were used for FWB program evaluation and user organisations found the results to be more inclusive, meaningful and relevant to their service needs and aspirations. To facilitate uptake of the program, researchers tailored the FWB intervention as a two- or three-day professional development course targeting the relevant health, education, employment, child protection, youth work, and business development support workforce.

The aforementioned outputs then contributed to some advances on the research program front. The fifth block outlines the direct initial impacts from doing the research. There was evidence of FWB meeting the needs as identified by FWB participants, including strengthening protective factors and contributing to people’s improved SEWB. Furthermore, the FWB approach to empowerment proved to be a good fit beyond Indigenous contexts—to be universally relevant and adaptable across cultures [28–31]. During FWB workshops, Aboriginal and Torres Strait Islander participants consistently called for Indigenous leaders and elders, managers of community organisations and parents, as well as non-Indigenous professionals working with Indigenous people, to be given the opportunity to attend FWB sessions so they could develop the SEWB capacity to better support those communities. The attributes and competencies promoted through FWB such as empathy, courage, kindness, non-judgement, patience, perseverance, and unconditional love, which participants valued, were identified as not just relevant to those perceived to be relatively disadvantaged, but as essential qualities for all humans irrespective of ethnicity, gender and social status. The evidence of FWB’s effect, combined with the research outputs, led to more requests for collaborative partnerships to attract funding to implement and evaluate FWB in new settings.

As a result of all the previous building blocks, the research impact can be measured in the recorded 5,405 people that have ultimately participated in the program, and the fact that the $2.3 million investment has led to consistent growth in the FWB intervention. Critically, what underpins all seven building blocks in the logic model are Aboriginal and Torres Strait Islander ethical research principles.

The arrow, pointing to the future, represents the changing context in which the research sits. The growing demand to demonstrate research impact is an opportunity for academic research to be accountable and beneficial to participants, but the process of documenting research impact is not straightforward. On the other hand, the environment has changed in that there are now more Indigenous health researchers who are influencing the research agenda and who open up new research partnerships to strengthen the evidence base.

### Family Wellbeing program uptake and reach

**Fig. 3:**
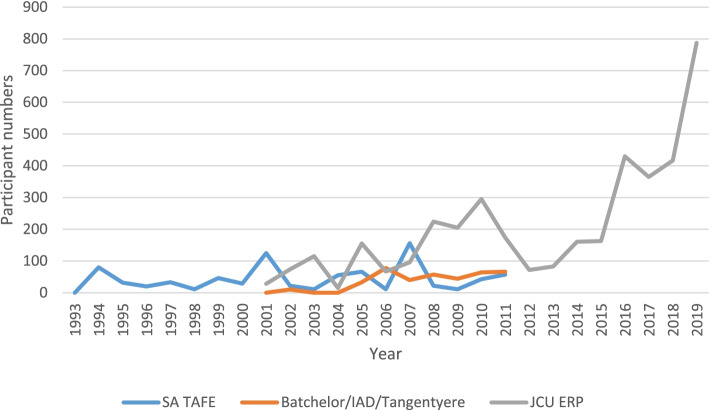
FWB participation over time and relative contribution of JCU-led research network

Figure 3 shows the variable but increasing annual number of FWB participants over the years. Of the 5,405 total participants for which there is definitive data, 537 (10%) participated through overseas pilot studies in Papua New Guinea (2009–2012), Timor-Leste (2016) and China (2016–2019).

When FWB began in 1993, TAFE SA was the sole FWB training provider and 467 people participated in the program for the first seven years. Following the pilot program evaluation in 1998–2000, and the dissemination of the findings, there was an increase in numbers as more people learnt about FWB. In Phase 2, during the 10-year ERP, 2,300 people participated through TAFE SA and two additional new providers, namely, Batchelor Institute of Indigenous Tertiary Education (NT) and the JCU-led research network. During this phase, the research informed the network that participants were benefitting even from limited attendance without being present for the entire 150-h course. As a result, two-day and three-day short courses (with follow-up peer support) were developed to make the program more accessible to people. Offering shortened courses further enabled uptake and spread of the program. In the third and final knowledge translation and impact phase, a total of 2,638 have participated through this JCU-network alone.

Looking at the increase in participation since 2001 (Fig. 3), it is unlikely that FWB would have achieved the levels of participation and impact that it did without the research and reporting of that research around Australia and the world. The dissemination of research publications and knowledge translation activities and resources including community reports, knowledge sharing forums and importantly, word of mouth (the “Indigenous grapevine”), all helped to spread the message about FWB program outcomes. People working in community organisations then learnt about the intervention and proceeded to support individuals to participate in FWB through one of the above-mentioned providers.

In the following section the statement of overall research impact claim and underpinning logic is supported by an examination of the nature and extent of the research investment.

### Family Wellbeing research related funding

Additional file 2 provides details of 16 FWB research-related grants totalling $2.308 million, received by the JCU-led FWB research network between 1998 and 2019. The initial small grant from Tangentyere Council, an Aboriginal community-controlled organisation, to evaluate their FWB program, became the catalyst for 23 years of FWB research with significant outputs and impacts. Grant amounts ranged from the initial $3,000 to $465,000 with the median being $82,000. The majority of grants were for a single year (9/16), followed by two years (6/16), and only one lasted three years. There were diverse funding sources – four grants from the National Health and Medical Research Council (NHMRC); three from Queensland Health; two from the Lowitja Institute and its predecessor Cooperative Research Centres; two from Apunipima Cape York Health Council; and several other health-related non-government organisations. The table also highlights that the research program relied on sourcing a variety of short-term funding in order to survive.

The next section provides a summary of the qualitative and quantitative evidence of SEWB outcomes of FWB. FWB program outcomes are highlighted as evidence of what the research found – and to argue that this evidence was of value to research users, and thus a vehicle to further program uptake.. The evidence in the following results speaks to the claim that FWB enhances the critical protective factors needed in contexts of intergenerational trauma and social disadvantage.

### Evidence of Family Wellbeing benefits

This section presents a summary of the reported SEWB benefits based on the results of the scoping review of nine evaluative papers (one qualitative, one quantitative and seven mixed methods) involving a total of 1,010 participants. The findings of these nine evaluative studies are reported elsewhere in detail (see Additional file 3 for references and study characteristics), but here a summary is provided to show how the evaluations facilitated uptake of the FWB program. Findings are divided into qualitative and quantitative evidence with a focus on the benefits in Indigenous settings. Relevant evaluative studies in non-Indigenous contexts are summarised in Additional file 3.

#### Reported qualitative social and emotional wellbeing outcomes

The evaluative studies brought to light some key SEWB benefits for Aboriginal and Torres Strait Islander participants of the program, and these findings resonate strongly with the range of protective factors outlined in the introduction. The findings are summarised into five themes below with the inclusion of quotes from the evaluations to demonstrate program outcomes. In addition, some important population-level outcomes are mentioned.

##### Personal growth

Participants reported on improved communication skills and the ability to better manage emotions. For example, a young Aboriginal man said; “I learned to use my voice, so I am a lot more confident to speak to people and crowds”; and a young Aboriginal woman reported that: “Before coming into this program, I bottled up all me emotions and feelings up but now I know that it’s better to talk to someone and know you’re not alone” (Central Coast NSW [49]).

Another woman had learned to understand and to manage her anger;

I feel very light… after bringing up some of the pains, you know? I think maybe all of us… It’s been quite a while since I’ve been angry, like after doing the course. I mean, I could get angry very easily… I thought I was nuts… whereas things I used to get angry about, I just don’t feel that anger any more (North Queensland [1]).

##### Increased confidence and resilience

These steps in personal growth contributed to increased self-confidence and esteem. An Aboriginal worker found that expressing her feelings gave her the space to think about how she could manage situations that arose for her; “…once you voice them then that gives the opportunity to think ‘well, what can I do about this?’ and maybe use the tools that are given to me through the program, that I can do something about it” (Victoria [40]). An Aboriginal community elder said she felt calmer and more confident; “I just sort of calmed down all the time… It just made you more confident in yourself, don’t sort of rush, panic or be frightened of things, making mistakes” (Victoria [40]).

##### Self-management

Participants took control of their SEWB in several ways including better management of mental health, alcohol use and gambling. This had flow-on benefits for other issues such as family relationships and financial stress;

Oh, yeah, things have changed. I guess I used to drink a lot and that and now I don’t drink that much. Now I’ve got money in the house, now that I’ve stopped drinking and I’ve got money to buy for the children. It’s because of the Family Wellbeing and the women’s group; it is those two things together (North Queensland [35]).

##### Building healthier and happier relationships

Participants spoke of building healthier and happier relationships with their families, teachers, and their communities. Participants learned how to better manage conflict in their families, and for some, this involved taking responsibility for their own behaviour;

Before when me and my wife used to fight, I used to get the rage and wanted to hit her…but now doing the FWB, I get to find other ways [of dealing with anger], plus [learning] how to deal with emotions and I find myself more at peace. (North Queensland [35]).

Also, participants learned to set boundaries and demand respect in their relationships; one woman told a story of her relationship with her nephew who;

…when drunk would come and punch walls and swear … I asked him to leave... he didn’t want to leave and argued he didn’t do or say anything wrong – so I wrote down the things that had upset me. [I] allowed him to come … once he had apologised… now he behaves sensibly because I stated ‘remember there’s the gate if you ever start your caper’ (Central Australia [27]).

##### Education and employment

Improved confidence and hope were evident as participants increasingly engaged in education, as demonstrated in this comment by a young Aboriginal man: “[the program] helped me get back into school. I'm now in a leadership program” (Central Coast NSW [49]). In Victoria, improvements in Aboriginal high school students’ school attendance were reported by both the students and the school principal in one region [40].

The effect of FWB participation was profound for this participant’s development;

I considered myself illiterate. I was pretty insecure. Once I did FWB I had more than I believed I had. Then I went to college and studied counselling. I had to write assignments. I hadn’t been to school since I was 14 (Central Australia [27]).

Participants also prepared for and engaged in employment. In Victoria, community members were reported to be getting “work ready”, for example going to a job agency or getting their drivers licence [40]. Several people managed to gain employment; “…one of the ladies who lives in [name withheld] Community House, she’s got a job …it is three hours preparing some food but it’s extra cash” (Victoria [40]).

##### Population level outcomes

The previous five FWB program outcomes point to the flow-on effects that individual improvements in SEWB and empowerment have for families, communities and networks. The community of Yarrabah (Far North Queensland), in particular, has achieved notable population-level results through its involvement with FWB. It was found to be one of only two Indigenous communities across Australia (Tiwi Islands being the other) that reduced high suicide rates in the past 20 years [50]. The evaluation noted that FWB research had made an essential contribution to this reduction.

#### Reported quantitative social and emotional wellbeing outcomes

The reported individual qualitative wellbeing benefits above are reflected in the results from the quantitative pre/three-month-post measures. As stated in the introduction, there can be a stigma placed on qualitative research of SEWB, even though it is this kind of research that captures compelling narratives about the effectiveness of wellbeing and empowerment interventions. To support these accounts, the FWB research program sought to quantitatively measure program outcomes as a way of increasing its profile, strengthening the FWB outcome evidence base, and assisting the spread of the program.

In one of the five Indigenous studies in this group, Kinchin et al. [51] examined four measuring tools on their sensitivity to detecting changes in emotional development, including communication, conflict resolution, decision making and life skill development, in child protection agency staff. Of the four tools assessed, the Growth and Empowerment Measure (GEM) responses on self-capacity, inner peace, strength, happiness and connectedness (questions 1–14) indicated a 17% positive change in the mean scores for FWB participants. With an *r* score of 17% and a *p-value* of < 0.001, the GEM proved to be the most sensitive tool in shedding light on how well participants coped with stress and demands on their time, as well as their perceptions of personal accomplishment and overall satisfaction with life [51].

Klieve et al. [49] reported improved participant wellbeing overall among two groups of vulnerable Aboriginal young men, with a highly significant reduction in psychological distress (*t* = 3.67, *df* = 12, *p* = 0.003) among the first group. The results were similar among the subsequent group with a significant decrease in the aggregated scores across the Kessler 5 scale (K5) items (*t* = 3.943, *df* = 47, *p* < 0.001, *d* = 0.5691). The study concluded that FWB might have given these young men an opportunity to enhance their SEWB and in turn had the potential to mitigate some of the costs associated with medical treatment and criminal interventions [49].

FWB delivered to Aboriginal health service workers who support users of methamphetamines was shown to significantly increase those workers’ GEM scores in life satisfaction (z = 2.25, p = 0.024) and inner peace (z = 2.25, p = 0.024). The effect sizes for all measures were large and positive (0.62–0.69), except for the self-capacity subscale where the effect size was small but still positive (0.16). In addition, participants reported improved wellbeing and feeling empowered in supporting users of methamphetamines and their families [40].

### Evidence of Family Wellbeing research utilisation or influence on program uptake

This section provides evidence of the utilisation of FWB research that led to decisions to implement the program in various contexts. The complete results of the review are presented in Additional file 4, however included here are the findings of three reflections by health and other human services managers on their respective decisions to use FWB in their organisations; and two national policy documents citing the research as evidence that FWB improves community functioning and SEWB, thereby helping to overcome Indigenous disadvantage.

In one of the three FWB user reflections, Baird [48] explained how a community-controlled health service formed a long-term relationship with the FWB research network to implement and evaluate the program in the community of Yarrabah. The research contributed to successful grant acquisitions which helped further the integration of the FWB program into the health service. Community members also became trained as researchers as part of empowering participants to conduct research in their own community.

Gabriel [52] described how, for a health service on the Central Coast in NSW, they first learnt of the program from reading the pilot evaluative study [27]. This led to uptake of FWB as part of their SEWB program for young Aboriginal men in the area. In Queensland, a child protection agency known as Act for Kids drew on FWB research to create a policy brief for senior management about the impact of the program [53]. As a result, a relationship developed between the organisation and the FWB research network which led to FWB becoming a practice framework across all its sites in Far North Queensland.

FWB research was cited in two nationwide reports. The Social Justice report by the Australian Human Rights Commission [54] described FWB “as an example in how to support communities to address complex problems by drawing on holistic healing methods which blend cultural renewal and spirituality with conflict resolution and other problem-solving skills”. Two government agency reports on overcoming Indigenous disadvantage cited FWB research papers as an example of a healing program that works [55, 56].

The evidence in the seven publications shows how the FWB research program influenced the thinking of both the primary user group i.e. the frontline service providers and, to some degree, policy makers.

## Discussion and conclusion

This article set out to explore the impact of the 23-year long FWB research program, and to identify the key enablers of research impact as well as the limitations of the impact data, as a way of planning for more targeted impact data collection into the future. The data supports an overall impact claim; that FWB research contributed to at least 5,405 participants, for which data exist, accessing the FWB program and potentially experiencing SEWB benefits as a result.

To assess impact of research on/by/for Aboriginal and Torres Strait Islander people, implementation science related models such as theories of change and service utilisation need to be modified to take account of a history of colonisation including research abuse and Indigenous self-determination. If research is intended to benefit Indigenous people, and for that matter any group of people, then the intended beneficiaries must decide what research is to be done, who does it, how it is done and how the results are used. This includes using the result for the benefits of those directly involved as well as translation to similar situations so as to avoid reinventing the wheel [57–59]. What this means in practice is that no matter where a research idea comes from, researchers have a responsibility to ensure that the research is informed by key Indigenous research ethical principles and values. This understanding allowed the creation of a FWB research impact logic model that maps out key enablers of research impact.

The first key enabler of research impact was that the research was user- or community-driven, in other words, the need for research came directly from Indigenous community organisations. The researchers were invited to evaluate the Aboriginal-developed FWB intervention and the results in turn generated interest and further demand, leading to deliberate efforts by researchers to develop a 10-year program of research in response to the growing demand [32, 33]. Rather than the usual case of researchers taking good ideas to partners and seeking support for a project, the researchers routinely sought to understand community partners’ or users’ interests and aspirations so they could consider how research might add value to such efforts [32].

The second key enabler of research impact was the long-term mutually beneficial partnerships approach adopted between researchers and research users. Researchers must be prepared to be involved for the long haul and be transparent about what is in it for all parties involved. By researchers undertaking community-driven research, there was evidence of user collaboration which helped in developing competitive grant applications; and producing papers and reports to meet academic expectations while at the same time supporting users to implement and evaluate the FWB program within their services. Taking this approach helped to clarify the roles and responsibilities of researchers vis-à-vis community partners and research users.

Thirdly, researchers created a body of knowledge that was inclusive of different research types. Generally, an applied research area is likely to achieve impact when it produces at least three types of outputs. The first is descriptive research that theorises or explains a phenomena, the causes and consequences of the issue, the extent or scale, and how the situation might be improved. Second is measurement research to qualitatively and quantitatively assess outcomes from policies, programs or services that are designed to improve the situation. Lastly, evaluative research outlines which solutions work for whom and under what conditions [32, 60, 61]. The publications resulting from the FWB research program incorporated all three research types. Critically, the outputs included Indigenous team members, especially those new to academic research, reflecting on their experiences as FWB facilitators and researchers in their own words. Utilising and valuing the different publication types, and their relative contributions to the overall body of knowledge, helped to overcome unproductive methodological wars regarding qualitative versus quantitative research. It promoted collaboration rather than competition among the research team as different members took the lead for different elements of the research depending on their expertise and interests [32].

A final enabling factor to research impact was that the FWB approach to empowerment was found to have applicability beyond Indigenous contexts, indicating its universality in promoting SEWB, and assisting in spreading the appeal of the program and associated research to a wider audience. There is a global trend for universities to embed the teaching of soft skills that foster student wellbeing and enables them to navigate rapidly changing social and global conditions. As social and political discourses become increasingly polarised, the approach of FWB is to emphasise individual and collective SEWB capacity and finding win–win solutions where people seek out common ground based on a common humanity. This widespread applicability has meant that FWB as a program has been incorporated into the curriculum for various university degrees in Australia, Papua New Guinea and China [28, 30, 31]. This also demonstrates the internationalisation of the associated research and impact [29].

In terms of limitations for this study, and for assessing research impact, analysis has been restricted to the use of retrospective data. Whilst the use of such data has provided the opportunity to demonstrate research impact and uptake of the program, it is acknowledged that the data was originally collected for other research purposes. Having said that, the application of Indigenous research principles allowed the researchers to collect data on issues that were identified by Aboriginal and Torres Strait Islander people, so it is from these measures which the authors of this article attempted to glean evidence of research impact. Into the future, what is required are clear evaluative indicators to measure the extent to which Indigenous research principles are applied in the research endeavour [14, 21]. More broadly, this study’s findings highlight the importance of a prospective approach to research impact assessment, supported by funding bodies, based on a logic model that guides evaluation of the concerned interventions [15]. Researchers need to create appropriate databases from the beginning and collect data well beyond the life of the project, even though it is acknowledged that such endeavours are rarely funded [14, 21].

The second limitation in assessing research impact is the short-term funding which constrained sustainable implementation and evaluation of FWB. For example, of the 56 sites where the FWB intervention and research had spread by 2011, the majority of those sites could not sustain it beyond the pilot phase [39]. Since most funding was ad hoc and relatively short-term (one to two years), this limited the capacity of the researchers to follow up with participants in the medium to long term. Despite this, a commitment to employing and supporting local Indigenous people as “community-based” researchers (in keeping with Indigenous research principles), not only increased employment and built research capacity but, importantly, it encouraged local ownership of the research and hence the capacity to use it to achieve desired outcomes [26, 48]. Furthermore, in 2021, an Australian anonymous philanthropist provided five-year funding for the FWB research network to support more consistent implementation and evaluation across the areas of education, employment, business development and health. This provides opportunities to strengthen not only the impact evaluation of the FWB intervention but also the research impact.

As this article has shown, determining if and how a particular piece of research translates into demonstrable impacts is not straight forward. As Whiteside et al. [25] put it, where knowledge translation and research impact begin and end is neither linear nor easily measurable. Besides, while FWB research is definitely important, it is only one factor among many that leads to ultimate impact, for example, the fact that the program is Aboriginal-developed may also account for its wide reach [62]. Therefore, it is not enough to talk about “research impact”, it is critical to highlight the relative contribution of research vis-à-vis other contributory factors to the impact claim. This is where research utilisation data is valuable because it explains, from the point of view of users, not only the extent to which the research influenced their decisions and actions, but importantly the impacts of such decisions. Without this information it is hard for researchers to show the pathways through which their research leads to particular impacts. Thus, collecting such data from the user’s point of view has significant potential to bridge the research impact attribution gap.

The FWB research impact exercise reinforced the view that assessing research impact is best approached as a “wicked problem” for which there are no easy fixes. It requires flexible, open-ended, collaborative learning-by-doing approaches to build the evidence base over time [21]. National research impact agendas by research funding bodies provide opportunities for research groups to proactively build the research impact knowledge base within and across their disciplines. Research groups can look back on their research projects over the past 10 years; identify projects they believe achieved impact; explore such impacts highlighting the challenges and opportunities; publish the findings in peer-reviewed journals, and use the lessons learned to inform their future research. Also, the body of research impact case studies being submitted for assessment in Australia, the UK, and other parts of the world, especially the publicly available ones, constitute potential data sources for research groups to systematically explore the challenges and opportunities of demonstrating impact through scoping and other reviews. For example, researchers can develop the knowledge base in terms of enablers and barriers of case study assessment to maximise engagement and impact within their disciplines, and they can also learn from available data how to produce high-quality impact case studies.

## Supplementary Information

**Additional file 1.** List of Family Wellbeing research outputs 1998- 2019

**Additional file 2.** Funding for Family Wellbeing program and research 1998-2020

**Additional file 3.** Systematic scoping review of evaluative studies

**Additional file 4.** Systematic scoping review of FWB research utilisation

## Data Availability

The datasets analysed during the current study are available in the James Cook University (ResearchOnline@JCU) https://researchonline.jcu.edu.au/ and the National Centre for FWB website https://family-wellbeing.squarespace.com/fwbprogram. All data generated or analysed during this study are included in this published article [and its Additional files].

## Abbreviations

AUWI: Australian Unity Wellbeing Index
CRC: Cooperative Research Centre
ERP: Empowerment Research Program
FWB: Family Wellbeing
GEM: Growth and Empowerment Measure
IAD: Institute for Aboriginal Development
JCU: James Cook University
K5: Kessler 5 scale
NHMRC: National Health and Medical Research Council
NSW: New South Wales
NT: Northern Territory
QLD: Queensland
SA: South Australia
SEWB: Social and Emotional Wellbeing
TAFE: Technical and Further Education
UK: United Kingdom

## Footnotes

1 In this article, the label “Indigenous” is used interchangeably with “Aboriginal” or “Aboriginal and Torres Strait Islander”.
