# Supplementary material for: “We are not stray leaves blowing about in the wind”: exploring the impact of Family Wellbeing empowerment research, 1998–2021
Source: Int J Equity Health. 2022 Jan 10;21:2. doi: 10.1186/s12939-021-01604-1 (PMC8744228; doi:10.1186/s12939-021-01604-1)
Supplement: Supplementary file 1 — Additional file 1. List of Family Wellbeing research outputs 1998- 2019 [file 12939_2021_1604_MOESM1_ESM.docx]

# Additional file 1

## List of Family Wellbeing research outputs 1998- 2019

- 16 grants
- 49 peer reviewed journal papers
- 19 reports
- 1 book and 4 book chapters
- 3 doctoral theses
- FWB knowledge sharing and translation resources including:
  - 8 Videos/DVDs; 1 FWB banner;
  - Quarterly newsletters from 2009-2013 and 2017-2018;
  - 1 policy brief;
  - a 2-day FWB Foundation Training package;
  - a 2-day FWB Facilitator Training package;
  - a James Cook University Master of Indigenous Studies/Master of Public Health subject
  - a La Trobe University Bachelor of Social Work subject
  - a collection of policy documents and evidence reviews citing FWB as promising and/or a that program that works based on our research.

These outputs are available online via for James Cook University (ResearchOnline@JCU) https://researchonline.jcu.edu.au/ and the National Centre for FWB website https://family-wellbeing.squarespace.com/fwbprogram.
