# Supplementary material for: “We are not stray leaves blowing about in the wind”: exploring the impact of Family Wellbeing empowerment research, 1998–2021
Source: Int J Equity Health. 2022 Jan 10;21:2. doi: 10.1186/s12939-021-01604-1 (PMC8744228; doi:10.1186/s12939-021-01604-1)
Supplement: Supplementary file 2 — Additional file 2. Funding for Family Wellbeing program and research 1998-2020 [file 12939_2021_1604_MOESM2_ESM.docx]

# Additional file 2

## Funding for Family Wellbeing program and research 1998-2020

| **Dates** | **Funding Body** | **Purpose** | **$ Amount** |
| --- | --- | --- | --- |
| 1998-99 | Tangentyere Council | Evaluate FWB training in Alice Springs | 3,000 |
| 2001 | Queensland Health via Gurriny Health and Yarrabah Men’s Group | FWB acceptability pilot with Indigenous men in Yarrabah | 5,000 |
| 2002 | National Suicide Strategy via Apunipima Cape York Health Council | FWB feasibility pilot in 2 Cape York Communities | 35,000 |
| 2002 | NHMRC | FWB follow-up evaluation in Alice Springs | 82,000 |
| 2003-5 | NHMRC | FWB pilot evaluation in 3 North Queensland communities | 465,000 |
| 2005-6 | CRCAH/NHMRC | Develop Growth and Empowerment Measure to evaluate Indigenous empowerment and wellbeing interventions | 127,000 (CRCAH) and 390,000 (NHMRC) |
| 2007-8 | Apunipima Cape York Health Council | FWB training in 10 Cape York communities | 340,000 |
| 2008 | Queensland Health | Mental health capacity building in 4 Queensland Indigenous communities | 100,000 |
| 2008 | Royal Flying Doctor Service | Mental health capacity building in 3 Gulf communities | 40,000 |
| 2009 | Australian Nurse Partnerships | FWB skills development among child and maternal health workers in 4 Indigenous health services across Australia | 82,000 |
| 2010 | Queensland Health /Northern Peninsular and Torres Strait Health | Wellbeing skills development among sexual health workers | 15,000 |
| 2013 | Healing Foundation via Act for Kids | Wellbeing skills development among Indigenous child protection workers in 5 remote North Queensland communities | 30,000 |
| 2016 | Timor-Leste Ministry of Health via Australian Embassy in Dili | Wellbeing soft skills development and team building among health leaders in Timor-Leste | 14,000 |
| 2016-17 | CRCAH/Lowitja Institute | Train and mentor frontline workers to integrate FWB into SEWB, child protection and youth services in north QLD, country Victoria and NSW Central Coast | 460,000 |
| 2018-19 | NHMRC Centre for Research Excellence - Integrated Quality Improvement | Support SEWB including FWB research translation and impact assessment | 120,000 |
| **Total** |  |  | **$2,308,000** |
