# Supplementary material for: “We are not stray leaves blowing about in the wind”: exploring the impact of Family Wellbeing empowerment research, 1998–2021
Source: Int J Equity Health. 2022 Jan 10;21:2. doi: 10.1186/s12939-021-01604-1 (PMC8744228; doi:10.1186/s12939-021-01604-1)
Supplement: Supplementary file 3 — Additional file 3. Systematic scoping review of evaluative studies [file 12939_2021_1604_MOESM3_ESM.docx]

# Additional file 3

## Systematic scoping review of evaluative studies

### Study characteristics

Over the last 23 years, the FWB research network has consistently collected evidence of the social and emotional wellbeing benefits of the FWB program. Of the nine included evaluative studies undertaken, one was qualitative in its methodology, one quantitative, and the other seven were pre- & three-month-post-workshop mixed-methods studies. A total of 1,010 persons participated across all the studies, ranging from 13 to 378 participants for each, with a median number of 92 participants. Six of the included studies were conducted in Indigenous Australian contexts while the other three were pilot studies in non-Indigenous contexts, designed to test the applicability of FWB in other cultural settings, and thereby to strengthen the evidence-base.

Of the six Indigenous-specific studies, five were conducted by the FWB researchers themselves while one was an independent report commissioned by the Healing Foundation to develop case studies of successful Indigenous suicide prevention (1). Of the five Indigenous studies conducted by the research team, one was a thematic synthesis of seven qualitative evaluative studies conducted across four communities in central Australia and north Queensland between 1998 and 2005, involving 148 adults and 70 school children (2) .

The other four studies included a quantitative impact evaluation involving 66 remote Indigenous child protection workers in north Queensland (3), and mixed methods impact evaluations with vulnerable Aboriginal young men (n=378) on the NSW Central Coast (4), SEWB workers in country Victoria (n=13) (5), and child protection workers in north Queensland (n=42) (6). The three non-Indigenous-setting pilots of the Aboriginal-developed FWB program involved social work students in Australia (n=64) (7), international trade students in China (n=29) (8) and 20 health leaders and managers in Timor-Leste (9). The quantitative data were collected through piloting different measures for their sensitivity, making it challenging for meta-synthesis. These measures included the Growth and Empowerment Measure (GEM) developed by the FWB research team (n=4 studies), Australian Unity Wellbeing Index (AUWI, n=4 studies), and Kessler (n=2).

### Table summarising nine Family Wellbeing program evaluative studies

|  | **1^st^ author, date** | **Study setting** | **Aims** | **Sample size** | **Methods** | **Social and emotional wellbeing outcomes** |
| --- | --- | --- | --- | --- | --- | --- |
| 1. | Tsey et. al., 2009 | FWB training in four Indigenous communities in Central Australia and North Queensland, Australia between 1998 and 2005. | To review and synthesise the reported FWB training outcomes across 7 discrete qualitative evaluative studies so as to develop a deeper understanding of the health and wellbeing impacts of empowerment in the Indigenous Australian context. | N = 218 (148 adults, 70 school children) | Qualitative thematic analysis of reported social and emotional wellbeing outcomes across the 7 studies . | Participants reported enhanced capacity to exert greater control over factors shaping their SEWB; a heightened sense of Indigenous and spiritual identity; increased respect for self and others; enhanced parenting; enhanced capacity to deal with substance abuse and violence.  Multilevel dimensions of empowerment were evidenced by changes made at the personal level that influenced other individuals and systems over time.  Highlighted was the consistency of the reported SEWB benefits across the different study settings. Also highlighted is the need to implement personal empowerment interventions as part of broader structural determinants of health such as employment, education and child/family support services in order to maximise outcomes. |
| 2. | Kinchin et. al, 2015 | FWB training for Indigenous child protection workers in five remote Indigenous communities, Far North QLD, 2013. | To enhance the capacity of remote Indigenous child protection workers to improve their own wellbeing so they can better support children and families with whom they work improve their wellbeing. | N = 66 employees | Quantitative survey pre/post (3 months after) using GEM (Growth and Empowerment Measure); AUWI (Australian Unity Wellbeing Index); K10 (Kessler psychological distress scale); WES (Workforce engagement survey). | GEM most sensitive to changes detected. Positive changes in communication, conflict resolution, decision making and life skill development (GEM *r*=17%, *p*<0.001; AUWI *r*=9%, *p*=0.004; WES *r*=5%, *p*=0.060; K10 *r*<1 %, *p*=0.715). |
| 3. | Prince, 2018 | Case study report commissioned by the Healing Foundation of suicide interventions in two Indigenous communities: Tiwi Islands in the Northern Territory and Yarrabah in north Queensland 2017. | Identify the success factors that underpin successful suicide prevention activity in two Indigenous Australia communities. | N = 31 Elders and community organization representatives from 2 communities | Interviews and group discussions | The report found Yarrabah to be one of two Indigenous communities Australia-wide to show evidence of successful suicide prevention over the past 20 years, Tiwi Island being the other community. The report identified a range of community-driven initiatives including FWB as contributory factors in Yarrabah.  The report identified a range of FWB specific outcomes as protective factors against suicide. These include: ‘reflective skills; hope and confidence; personal healing and improved ability to control destructive behaviours and emotions; prevention and management of domestic conflict and more positive family relationships; safe environments to voice pain and enhance the ability to explore feelings and attitudes; reduced levels of alcohol consumption and conflict; interest in sharing experiences in dealing with suicide with other communities’ (pp 26-27).  While suicide is still a concern, both communities reported being more prepared in dealing with it when it happens. |
| 4. | Whiteside et al, 2018 | FWB training for Indigenous SEWB workers at Mallee District Aboriginal Services (MDAS) in rural Victoria 2017. | Determine the feasibility of FWB in enhancing the capacity of Indigenous SEWB workers to improve their wellbeing so they can better support users of methamphetamines (ICE) and their families. | N = 13 (11 employees, 1 researcher, 1 social work student) | Pre/post (3 months after) mixed methods using open-ended qualitative questions and Growth and Empowerment Measure (GEM) | Qualitative: Participants reported improved wellbeing and feeling empowered in supporting users of methamphetamines and their families.  Quantitative: Significantly higher scores in the post-test life satisfaction scale (*z*=2.25, *p*=.024) and inner peace subscale of GEM (*z*=2.25, *p*=.024).  Effect sizes for all measures except for the self-capacity subscale were large and positive (0.62-0.69), the effect size for the self-capacity subscale was small but still positive (0.16). |
| 5. | Klieve et al, 2019 | FWB training for disenfranchised young Aboriginal men at risk of disengaging from school and/or entering the juvenile justice in NSW Central Coast (3 waves of training between 2014 and 2018). | Assess the SEWB impacts among the training participants. | N = 378 (327 program participants, 51 participant supporters) | Pre/post (3 months after) mixed methods using open-ended qualitative questions; K5 and ASSIST (Alcohol, Smoking and Substance Involvement Screening Tool) quantitative measures. | Qualitative: Enhanced personal confidence, self-esteem and resilience in participants; ability to control emotions; reduction in drug and alcohol use; improved relationship skills and relationships; planning for a better life; and engagement in learning and employment.  Quantitative: Period 1: highly significant reduction in psychological distress across K5 items (*t*=3.67, *df*=12, *p*=.003) with very strong effect size (*d*=1.02). Period 2: similar effect with a significant decrease in the aggregated scores across the K5 items (*t*=3.943, *df*=47, *p*< .001, *d*=0.5691), with the effect size indicating a medium to high effect. Period 3: alcohol and drug use identified in 84% of participants. |
| 6. | Onnis et al, 2020 | Train and support Indigenous child protection workers to integrate FWB as practice framework within their services, north Queensland, 2016-18 | Understand factors that enabled the successful integration of FWB program into the practices of a child and family support service. | N = 42 employees | Pre/post (3 months after) mixed methods using qualitative open-ended questions and quantitative K5 and AUWI measures | A conceptual model of enablers of successful FWB integration that include: Indigenous leadership, common language, build capacity, personal wellbeing*, customisation of the program, and relationships.  *With regards to wellbeing, participants reported increased personal growth and development; enhanced capacity to facilitate the program; and improved relationships within and across organisations.  Positive overall assessment of experiences (60-90%), low levels of psychological distress in more than half of participants. One very low score (27.5%/17) improved moderately after completion of training. |
| 7. | Whiteside et al, 2017 | Integrate FWB into an undergraduate social work curriculum 2016 | Assess the impact of FWB on student wellbeing | N = 64 students | Pre/post mixed methods (3 months) using qualitative open-ended questions and quantitative GEM and AUWI | Qualitative: Participants reported improved personal wellbeing; improved social competencies, including self-awareness, self-confidence, building relationships, and managing stress; increased engagement with their university; and increased professional knowledge.  Quantitative: A statistically significant change over time in the item “I feel knowledgeable about things that are important” (*Z*=−3.193, *p*<.001) potentially influenced the outcomes for overall GEM14 (*Z*=−2.120, *p*=0.034), and the GEM14 “other” subscale (*Z*=−2.496, *p*<.01).  For the whole sample, there was a small effect size for the GEM14 (*r*=0.27), and a medium effect size for the GEM14 “other” subscale (*r*= 0.39); for the AUWI effect size was medium (*r*=0.31). |
| 8. | Tsey et al, 2018 | FWB training for health managers and leaders from across the Timor-Leste Ministry of Health to facilitate wellbeing and leadership development, 2016 | Examine the acceptability and feasibility of the Aboriginal-developed FWB in the context of leadership soft skills development among Timor-Lester health managers and leaders. | N = 20 program participants | An exploratory mixed-methods design was adopted, where quantitative impact data were collected at the end of the workshop to complement qualitative workshop evaluation data. All 20 workshop participants answered online AUWI and MindTools Leadership questionnaires at the end of the five-day workshops to provide data on the level of their leadership skills before and after the workshop, their personal well-being before and after the training, and workshop evaluation feedback. | Qualitative: Participants reported increased self-awareness and feeling well prepared for their leadership roles.  Quantitative: Significant increase in perceived pre-program wellbeing scores (7.15-8.20) (*p*<.001), strong effect size in 7/8 (*d*>0.7), medium effect size in 1/8 (*d*=0.622).  Highly significant improvement in understanding of leadership skills (*t* value=5.146, *p*<.003). Highly significant scores in emotional intelligence (*t* value: 7.855, *p*=.025) and in 3/5 transformational leadership domains (*t* values 4.333, 4.925, 5.894*, p*=.000). |
| 9. | Yan et al, 2018 | FWB as soft skills leadership training for undergraduate International Trade students at Shenyang University of Chemical Technology (SUCT) in north-east China. | Assess the impact of FWB on student wellbeing and leadership soft skills. | N = 29 students | An exploratory mixed-methods pre/post (3 months) design using qualitative open-ended questions and quantitative AUWI and MindTools online survey. | Qualitative: Students reported increased confidence; self-awareness; class harmony; improved relationships; increased positive attitude toward others; improved communication skills; and a deeper understanding of the leadership concept.  Quantitative: Statistically significant higher overall wellbeing index score (*p*<0.001) than before the training.  Effect size overall large (0.85) with medium to large score individually (0.66-0.85).  Improved overall leadership skills post score of 64.90 (pre-53.24) with a *p* value of < 0.001. |

## References

1. Prince J, Jeffrey N, Baird L, Kingsburra S, Tipiloura B, Dudgeon P. Stories from community - How suicide rates fell in two Indigenous communities. Canberra, Australia: Aboriginal & Torres Strait Islander Healing Foundation; 2018.

2. Tsey K, Whiteside M, Haswell-Elkins M, Bainbridge R, Cadet-James Y, Wilson A. Empowerment and Indigenous Australian health: a synthesis of findings from Family Wellbeing formative research. Health Soc Care Community. 2009;18(2):169–79.

3. Kinchin I, Jacups S, Tsey K, Lines K. An empowerment intervention for Indigenous communities: an outcome assessment. BMC Psychol [Internet]. 2015 Aug 21 [cited 2021 May 11];3(1). Available from: https://www.ncbi.nlm.nih.gov/pmc/articles/PMC4546092/

4. Klieve H, Cheer K, Whiteside M, Baird L, MacLean S, Tsey K. “A safe haven to support me”: an evaluation report on the Central Coast Family Wellbeing Program [Internet]. Cairns, Queensland: Cairns Institute; 2019 [cited 2020 Aug 19]. Available from: https://researchonline.jcu.edu.au/59155/

5. Whiteside M, MacLean S, Callinan S, Marshall P, Nolan S, Tsey K. Acceptability of an Aboriginal Wellbeing Intervention for Supporters of People Using Methamphetamines. Australian Social Work. 2018 Jul 3;71(3):358–66.

6. Onnis L-A, Moylan R, Whiteside M, Klieve H, Smith K, Tsey K. Integrating the Family Wellbeing Program Into Practice: A Conceptual Model. Australian Social Work. 2020 Oct 1;73(4):435–48.

7. Whiteside M, Bould E, Tsey K, Venville A, Cadet-James Y, Morris ME. Promoting Twenty-first-century Student Competencies: A Wellbeing Approach. Australian Social Work. 2017 Jul 3;70(3):324–36.

8. Yan L, Yinghong Y, Lui SM (Carrie), Whiteside M, Tsey K. Teaching “soft skills” to university students in China: the feasibility of an Australian approach. Educational Studies. 2019 Mar 4;45(2):242–58.

9. Tsey K, Lui SM (Carrie), Heyeres M, Pryce J, Yan L, Bauld S. Developing Soft Skills: Exploring the Feasibility of an Australian Well-Being Program for Health Managers and Leaders in Timor-Leste. SAGE Open. 2018 Oct 1;8(4):2158244018811404.
