# Supplementary material for: “We are not stray leaves blowing about in the wind”: exploring the impact of Family Wellbeing empowerment research, 1998–2021
Source: Int J Equity Health. 2022 Jan 10;21:2. doi: 10.1186/s12939-021-01604-1 (PMC8744228; doi:10.1186/s12939-021-01604-1)
Supplement: Supplementary file 4 — Additional file 4. Systematic scoping review of FWB research utilisation [file 12939_2021_1604_MOESM4_ESM.docx]

# Additional file 4

## Systematic scoping review of FWB research utilisation

### Table summarising seven accounts of Family Wellbeing research utilisation

|  | **1^st^ author, date** | **Description** | **Evidence of research utilisation** | **Reported outcomes** |
| --- | --- | --- | --- | --- |
|  | Australian Human Rights Commission, 2011(1) | Social Justice report regarding the experience and exercise of human rights by Aboriginal and Torres Strait Islanders. | The FWB program, as it was utilised in Yarrabah to address the high rates of suicide in their community, was highlighted as an example in how to support communities to address complex problems by drawing on holistic healing methods which blend cultural renewal and spirituality with conflict resolution and other problem-solving skills (p. 145-149).  The FWB research papers cited in the report included McEwan & Tsey (2009) (2), Tsey et al. (2009) (3), Tsey et al. (2003) (4), and Whiteside et al. (2006) (5). | National recognition of FWB through research gave the program credibility in the eyes of potential user organisations. |
|  | Baird, 2019 (6) | A personal account of a former CEO of a new Indigenous community health service in Yarrabah who invited FWB researchers to help introduce the program into the community | Father Les Baird reached out to Komla Tsey in 2000 to find out more about his research and discuss a possible collaboration between the university and Yarrabah to address the high suicide rates in the community. After initial consultation it was agreed upon developing a social, emotional and spiritual health program that complemented Queensland Health’s medical services in the community, which was later transformed into a community-controlled comprehensive primary health care service. At the start of the research collaboration, the health service had only three employees (the CEO included) and provided no services. FWB and other research grants allowed the new service to employ and train four community-based researchers (2 male and 2 female) to help pilot FWB with a range of community groups. In response to the positive outcomes, the Department of Health and Human Services (DOHA) provided permanent funding for the four positions, now designated as Men and Women Health Workers. Today, the service employs just over 100 staff, 80% from the local community. Participatory Action Research processes empowered community members as they were actively involved in the research concerning their community, as opposed to being researched by outsiders. | Improved SEWB among a variety of workers and community members who participated in the FWB workshops; certification of FWB facilitators (from the community) to deliver the program to a variety of community groups; a national suicide prevention grant awarded to Yarrabah community to share its FWB and Men’s Group experience with three other Indigenous communities in Queensland who were facing issues relating to suicide; empowered the community to build their own health service including taking over QLD Health medical services as part of comprehensive primary health care |
|  | Moylan and Drew, 2017 (7) | Newsletter story by Act for Kids, a child abuse prevention service, reflecting on the role FWB research played in the uptake and integration of the program into their services. | In response to Act for Kids-JCU FWB pilot training, the Act for Kids cultural mentor and the training coordinator prepared a presentation to convince the senior management team about the benefits of the FWB program and how it could assist their staff and the families with whom they work. Not knowing how mainstream clinicians in the senior management team would perceive the FWB program, they decided to ‘rely heavily’ on 10 years of FWB research summarised in the form of a Lowitja Institute policy brief showing that ‘FWB delivered in different sites revealed that participation in the program enhanced people’s capacity to take control of their lives. And at an individual level, empowerment manifested through attributes such as hope, goal setting, communication skills, empathy, a strong desire to help others, perseverance and a belief that the social environment can change’. | The presentation to the senior management team was well received and the team began to gather ideas on how FWB could be incorporated in their day-to-day operations. Since then, Act for Kids applied for a $50K grant from QLD Health and in collaboration with JCU has successfully integrated FWB as practice framework for their five Safe Houses in Napranum, Aurukun, Pormpuaaw, Kowanyama and Doomadgee. |
|  | Gabriel, 2017 (8) | Newsletter story about how NSW Central Coast Primary Care (CCPC) service adopted FWB as a program that could support SEWB of young Aboriginal men in the region | During literature searches and discussions of programs that may fit, the FWB research came up frequently. It was the evaluation paper by Tsey and Every (9) that prompted the service to get in contact with Prof Tsey initially. This was followed by FWB researchers training and supporting local people to implement the program targeting young Aboriginal men at risk of dropping out school and/or entering the juvenile justice system. | Between 2012 and 2017, the service ran 22 programs with young Aboriginal men inclusive of several programs within the Frank Baxter Juvenile Justice Centre. Four intensives ‘train the trainer’ groups have been run with Aboriginal staff and community members. One pilot group has been run with young Aboriginal women. A total of 378 (327 program participants, 51 participant supporters) benefited from the research collaboration. (10,11) |
|  | Closing the Gap Clearinghouse (AIHW, AIFS), 2013 (12)  Productivity Commission, 2016 (13) | Annual reports by the Productivity Commission to the Australian Parliament on ‘what works to overcome Indigenous disadvantage’ designed to track progress towards closing the health gap. | Citing FWB research papers (3,9), the 2013 and 2016 reports described FWB as a *“cultural healing program”* and a *“thing that works”* to promote Indigenous community functioning, 2013 p.26; 2016 p.3171. | National recognition of FWB through research gave the program credibility in the eyes of potential user organisations |
|  | McCalman, 2013; McCalman et al., 2018 (14,15) | A theoretical model of program transfer based on interviews with 18 Aboriginal and non-Aboriginal research respondents who had been active in transferring the program.  A historical analysis of the conditions that enabled the spread of FWB to at least 60 geographical sites across Australia over 24 years | Organisations transferred the program by using it as a vehicle for supporting inside-out empowerment.  The research evidence from FWB empowerment interventions build credibility for the program and played a key role in the transfer and spread of the program. | Program transfer occurred through a multi-levelled process of embracing relatedness which included relatedness with self, others, and structural conditions; all three were necessary at both individual and organisational levels.  Program spread was influenced by a range of factors including the fact that the program was Aboriginal-developed; government policies; availability of funding and support; Aboriginal leadership including facilitator capability; informal support networks; and research evidence. |
|  | Whiteside et al., 2021 (16) | A pilot FWB research impact case study based on the Australian Research Council impact framework, Mildura, country Victoria, 2016-2019. | Research by Mallee District Aboriginal Services (MDAS) and Melbourne University alcohol researchers into methamphetamines or ice use in regional Victoria recommended a need for an appropriate family support program to build the capacity of workers and families to better support people to recover from ice use. The lead alcohol researcher happened to be a colleague of FWB researcher Mary Whiteside and hence was familiar with FWB which she considered potentially a ‘good fit’ Indigenous family support program. The alcohol researcher collected information on FWB from Mary which she passed on to MDAS. In response, MDAS managers invited Mary to pilot FWB with workers resulting, after two years of collaborative work, in an eventual decision by MDAS to integrate FWB as a whole of service model. | Examples of services that integrated FWB included MDAS early years family support service; gambling harm prevention program; justice rehabilitation centre; child friendly school activities; youth mental health strategy; and the Koori women’s diversion program.  Capturing research impact is important but it can be complex. Social research is rarely mechanistic, and more often complicated and messy with multiple strands and partners. This is more so in the case of intervention research. Where does knowledge translation and research impact begin and end? Did impact commence with the start of the Family Wellbeing pilot study? Or when the user organisation had taken ownership of the process and integrated it as their whole-of-service practice framework? The study recommended more work to develop the methods and frameworks required for social researchers to effectively capture impact. |

## References

1. Australian Human Rights Commission. Social Justice Report 2011 [Internet]. 2011. Available from: https://humanrights.gov.au/sites/ default/files/content/social_justice/sj_report/sjreport11/pdf/sjr2011.pdf

2. McEwan A, Tsey K. The role of spirituality in social and emotional wellbeing initiatives: The Family Wellbeing Program at Yarrabah [Internet]. 2009. (Cooperative Research Centre for Aboriginal Health Discussion Paper No 7). Available from: https://www.lowitja.org.au/page/services/resources/family-and-community-health/families/role-of-spirituality-in-social-and-emotional-wellbeing

3. Tsey K, Whiteside M, Haswell-Elkins M, Bainbridge R, Cadet-James Y, Wilson A. Empowerment and Indigenous Australian health: A synthesis of findings from Family Wellbeing formative research. Health Soc Care Community. 2009;18(2):169–79.

4. Tsey K, Whiteside M, Deemal A, Gibson T. Social Determinants of Health, the ‘Control Factor’ and the Family Wellbeing Empowerment Program. Australas Psychiatry. 2003 Oct 1;11(1_suppl):S34–9.

5. Whiteside M, Tsey K, McCalman J, Cadet-James Y, Wilson A. Empowerment as a framework for Indigenous workforce development and rrganisational change. null. 2006 Dec 1;59(4):422–34.

6. Baird L. The solution to Indigenous suicide crises lies in listening to Aboriginal people. Overland [Internet]. 2019 Jun 24 [cited 2020 May 11]; Available from: https://overland.org.au/2019/06/the-solution-to-indigenous-suicide-crises-lies-in-listening-to-aboriginal-people/

7. Moylan R, Drew V. It’s not about reinventing the wheel, it’s about making the wheel turn. Family Wellbeing newsletter [Internet]. 2017;(3). Available from: https://www.cairnsinstitute.jcu.edu.au/fwb-newsletter/

8. Gabriel Z. Family Wellbeing, Central Coast NSW… the story so far. Family Wellbeing newsletter [Internet]. 2017;(3). Available from: https://www.cairnsinstitute.jcu.edu.au/fwb-newsletter/

9. Tsey K, Every A. Evaluating Aboriginal empowerment programs: the case of Family WellBeing. Australian and New Zealand Journal of Public Health. 2000;24(5):509–14.

10. Whiteside M, Klieve H, Millgate N, Webb B, Gabriel Z, McPherson L, et al. Connecting and strengthening young Aboriginal men: A Family Wellbeing pilot study. Australian Social Work. 2016 Apr 2;69(2):241–52.

11. Klieve H, Cheer K, Whiteside M, Baird L, MacLean S, Tsey K. “A safe haven to support me”: An evaluation report on the Central Coast Family Wellbeing program [Internet]. Cairns, Queensland: Cairns Institute; 2019 [cited 2020 Aug 19]. Available from: https://researchonline.jcu.edu.au/59155/

12. Closing the Gap Clearinghouse (AIHW & AIFS). What works to overcome Indigenous disadvantage: key learnings and gaps in the evidence [Internet]. Canberra: AIHW; 2013 [cited 2021 May 14]. Available from: https://www.aihw.gov.au/reports/indigenous-australians/works-to-overcome-indigenous-disadvantage-2011/contents/table-of-contents

13. Productivity Commission. Overcoming Indigenous Disadvantage: Key Indicators 2016 [Internet]. Canberra: Productivity Commission; 2016 [cited 2021 May 16]. Available from: https://www.pc.gov.au/research/ongoing/overcoming-indigenous-disadvantage/2016

14. McCalman J. The transfer and implementation of an Aboriginal Australian wellbeing program: a grounded theory study. Implement Sci. 2013 Oct 31;8:129.

15. McCalman J, Bainbridge R, Brown C, Tsey K, Clarke A. The Aboriginal Australian Family Wellbeing program: A historical analysis of the conditions that enabled its spread. Front Public Health. 2018 Mar 1;6:26–26.

16. Whiteside M, Thomas D, Griffin T, Stephens R, Maltzahn K, Tsey K, et al. Capturing research impact: The case study of a community wellbeing research partnership. Australian Social Work. 2021 Mar 17;0(0):1–12.
